# ArtinM Cytotoxicity in B Cells Derived from Non-Hodgkin’s Lymphoma Depends on Syk and Src Family Kinases

**DOI:** 10.3390/ijms24021075

**Published:** 2023-01-05

**Authors:** Bruno Rafael Barboza, Sandra Maria de Oliveira Thomaz, Airton de Carvalho Junior, Enilza Maria Espreafico, Jackson Gabriel Miyamoto, Alexandre Keiji Tashima, Maurício Frota Camacho, André Zelanis, Maria Cristina Roque-Barreira, Thiago Aparecido da Silva

**Affiliations:** 1 Laboratory of Immunochemistry and Glycobiology, Department of Cell and Molecular Biology and Pathogenic Bioagents, Ribeirão Preto Medical School, University of São Paulo (FMRP/USP), Ribeirao Preto 14049-900, SP, Brazil; 2 Laboratory of Cell and Molecular Biology of Cancer, Department of Cell and Molecular Biology and Pathogenic Bioagents, Ribeirão Preto Medical School, University of São Paulo (FMRP/USP), Ribeirao Preto 14049-900, SP, Brazil; 3 Department of Biochemistry, Paulista School of Medicine, Federal University of São Paulo (EPM/UNIFESP), Sao Paulo 04021-001, SP, Brazil; 4 Functional Proteomics Laboratory, Department of Science and Technology, Federal University of São Paulo (ICT-UNIFESP), São José dos Campos 04021-001, SP, Brazil; 5 Laboratory of Immunotherapy of Invasive Fungal Infections, Department of Cell and Molecular Biology and Pathogenic Bioagents, Ribeirão Preto Medical School, University of São Paulo (FMRP/USP), Ribeirao Preto 14049-900, SP, Brazil

**Keywords:** ArtinM, lectin, apoptosis, murine and human B cells, leukemia

## Abstract

Receptors on the immune cell surface have a variety of glycans that may account for the immunomodulation induced by lectins, which have a carbohydrate recognition domain (CRD) that binds to monosaccharides or oligosaccharides in a specific manner. ArtinM, a D-mannose-binding lectin obtained from *Artocarpus heterophyllus*, has affinity for the N-glycans core. Immunomodulation by ArtinM toward the Th1 phenotype occurs via its interaction with TLR2/CD14 N-glycans on antigen-presenting cells, as well as recognition of CD3γ N-glycans on murine CD4+ and CD8+ T cells. ArtinM exerts a cytotoxic effect on Jurkat human leukemic T-cell line and human myeloid leukemia cell line (NB4). The current study evaluated the effects of ArtinM on murine and human B cells derived from non-Hodgkin’s lymphoma. We found that murine B cells are recognized by ArtinM via the CRD, and the ArtinM stimulus did not augment the proliferation rate or production of IL-2. However, murine B cell incubation with ArtinM augmented the rate of apoptosis, and this cytotoxic effect of ArtinM was also seen in human B cell-lines sourced from non-Hodgkin’s lymphoma Raji cell line. This cytotoxic effect was inhibited by the phosphatase activity of CD45 on Lck, and the protein kinases of the Src family contribute to cell death triggered by ArtinM.

## 1. Introduction

Glycosylation is a phenomenon in the post-translational modification of proteins [1]. The linkage of glycans (monosaccharides or oligosaccharides) to proteins and lipids gives rise to glycoconjugates [2]. The attached glycans may account for biological activities in different cell types [3]; for example, innate and adaptive immune cells exhibit a panel of receptors for distinct classes of glycans [4,5,6,7,8], and their binding leads to cell activation and immunomodulation mediated by cytokine release and/or cell differentiation [9,10,11,12]. In addition, glycoproteins on the cell surface can mediate apoptosis of immune cells [13]. Glycan recognition by lectins may trigger biological responses [3]. Lectins are heterogeneous proteins whose carbohydrate recognition domain (CRD) reacts with mono- or oligosaccharides in a specific, reversible, and noncovalent manner. Studies on the interactions between lectins and glycoconjugates [14,15,16] have revealed their ability to modulate immune responses [17,18].

Our group previously reported the immunomodulatory activity of ArtinM, a D-mannose-binding lectin obtained from the seeds of *Artocarpus heterophyllus*. ArtinM is structurally organized as a homotetramer formed by 16-kDa non-glycosylated subunits, each polypeptide chain containing a CRD with an affinity for Manα1–3 [Manα1–6] Manβ1–4, which constitutes the N-glycans core [19,20,21]. Immunomodulation of the Th1 phenotype by ArtinM occurs via its interaction with TLR2/CD14 N-glycans on antigen-presenting cells, which induces IL-12 production [8,22]. In addition, ArtinM interacts with CD3γ N-glycans on murine CD4^+^ and CD8^+^ T cells, contributing to Th1 response and T cell proliferation [23]. ArtinM targets mast cells and neutrophils via interactions with FcεRI (high-affinity IgE receptor) [24] and CXCR2 (CXC-chemokine receptor type 2) [25], respectively. Recently, we demonstrated the capacity of ArtinM to induce murine B cell activation, detected by the augmented production of IL-17 and IL-12p40 cytokines, a response that does not depend on lectin binding to TLR2/CD14 [26]. In contrast, ArtinM exerts a cytotoxic effect on Jurkat human leukemic T-cell line [23] and human myeloid leukemia cell line (NB4) [27]. These findings indicate that ArtinM induces distinct biological activities in several cell types, including tumor cells, via carbohydrate recognition.

B cells originate from hematopoietic stem cells (HSCs) in the bone marrow (BM) in adulthood [28]. HSC differentiation into mature B cells occurs at different stages, pro-B, pre-B, and immature B cells, characterized by the expression of cell surface-specific markers and successive rearrangement of the immunoglobulin (Ig) heavy (H) and light (L) gene segments [29]. B lymphocytes reach the final stage when Ig-kappa and/or Ig-lambda L chains rearrange over time during antigen-independent development [30]. The subdivision of B cells into B-1 and B-2 cells is based on their phenotype and functional properties. The two subpopulations are distinct in their ontogeny, anatomical distribution, and immune response [31,32,33]. B-1 cells are usually found in the peritoneal cavity [34] and are the leading producers of natural polyreactive antibodies [35]. The B-2 subpopulation comprises spleen marginal zone B cells (B-Mz) and follicular B cells (B-Fo) from the spleen and lymph node follicles [36]. The regulation of growth, differentiation, and function of B cells are regulated by B cell receptor (BCR) that is composed of a membrane immunoglobulin molecule (antigen recognition) associated with a heterodimer of proteins CD79A and CD79B (signaling transduction domain). The signaling triggered by BCR can induce a proliferation, differentiation, survival, anergy or apoptosis; and members of the Src family kinases (Lyn, Fyn or Blk) that act in the phosphorylation of CD79A and CD79B for the recruitment of Syk molecule to propagate the BCR signal [37]. This event is accounted for several cellular responses in normal and malignant B cells [38]. In addition, CD45 phosphatase activity acts in the regulation of Src activation by dephosphorylation of Src phosphotyrosine 527 [39].

B-Fo cells are essential for structuring the germinal centers (GCs) of follicles, where antibody production, affinity maturation, and diversity occur [40]. Although GC reactions are strictly regulated, regulator genes are susceptible to mutations that favor malignant transformation of B cells and occurrence of lymphomas [41]. Most lymphomas resulting from GC reactions are non-Hodgkin’s lymphoma (B-NHL) types [42,43]. Burkitt lymphoma is a highly aggressive B-NHL subtype [44]. The molecular signature of all BL subtypes involves chromosomal rearrangement with translocation of the c-MYC gene (8; 14) into the immunoglobulin heavy chain locus [45,46]. Several other genes have been reported as potential inductors of BL tumorigenesis, such as those involved in the BCR and PI3K/AKT signaling pathways, apoptosis, epigenetic regulation, and G protein-coupled receptors [47].

In this study, we report new molecular mechanisms used by ArtinM to induce apoptosis in B-NHL cells. Herein, we demonstrated that ArtinM at low concentrations increased the mitochondrial activity of murine B cells without causing cell proliferation. We also verified apoptosis in murine B cells and the cytotoxic effect of ArtinM in human B cells derived from NHL. Carbohydrate recognition accounts for Raji and Daudi cell apoptosis induced by ArtinM in a manner dependent on protein kinases of the Syk and Src families and phosphatase activity of CD45. Quantitative proteomics revealed low levels of proteins associated with cell proliferation and survival regulation in ArtinM-stimulated Raji cells. Our findings provide new directions to be considered in delineating strategies for cancer therapy based on carbohydrate recognition.

## 2. Results

### 2.1. ArtinM Increases the Mitochondrial Activity of B Cells without Inducing Cell Proliferation

**Figure 1:**
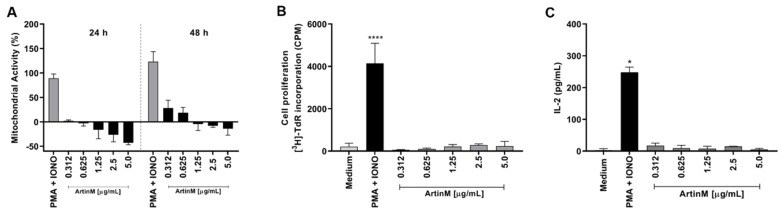
Increased mitochondrial activity of murine B cells induced by ArtinM is not accompanied by B cell proliferation or IL-2 production. B cells were purified from a spleen cell suspension obtained from C57BL/6 mice, seeded in 96-well microplates (5 × 10^5^ cells/well), and incubated with ArtinM at different concentrations (**A**–**C**). A mixture of Phorbol-12-myristate-13-acetate (PMA; 50 ng/mL) and Ionomycin (IONO; 1 µM) was used as a positive control for cell activation, and medium alone was used as a negative control (Medium). (**A**) Following 24 or 48 h incubation with ArtinM, 3-(4,5-dimethyl-2-thiazolyl)-2,5-diphenyl-2H-tetrazolium bromide (MTT; 50 µg/mL) was added to the cells, and mitochondrial activity was estimated by MTT reduction and expressed as a percentage calculated from the ratio between the absorbance of stimulated and non-stimulated B cells. (**B**) Following 48 h incubation with ArtinM, the proliferative rate of murine B cells was calculated according to thymidine incorporation ([^3^H]-TdR) (0.5 µCi/well); results are expressed as count per minute (CPM). (**C**) IL-2 levels in the supernatant of ArtinM-stimulated B cells, for 48 h, were measured using ELISA; the values are expressed in pg/mL. Significant differences compared to the medium are shown by *, *p* < 0.05 and ****, *p* < 0.0001.

The immunomodulatory activity of ArtinM on different innate immune cells and T cells has been well studied, whereas the effect of ArtinM on B lymphocytes has not been sufficiently explored. In this study, we first assessed the effects of ArtinM on purified murine splenic B cells. Mitochondrial activity was measured using the MTT assay 24 and 48 h after incubation with ArtinM (0.312–5 µg/mL) (Figure 1A). Mitochondrial activity did not increase after 24 h incubation. However, at 48 h, stimulation with 0.312 and 0.625 µg/mL ArtinM increased the mitochondrial activity of B cells by 28.5% and 18.7%, respectively, in comparison to unstimulated cells (Figure 1A). Because augmentation of mitochondrial activity is frequently related to cell proliferation, the 48 h ArtinM-stimulated cells were evaluated using the tritiated thymidine ([^3^H]-TdR) incorporation assay, and IL-2 production was measured using enzyme-linked immunosorbent assay (ELISA). Compared with unstimulated cells (medium), B cells stimulated with any ArtinM concentration (0.312–5 µg/mL) did not show increased [^3^H]-TdR incorporation (Figure 1B), or IL-2 levels (Figure 1C). Therefore, ArtinM stimulated the mitochondrial activity of murine B cells without inducing cell proliferation.

### 2.2. ArtinM Induces Apoptosis of Murine B Cells

**Figure 2:**
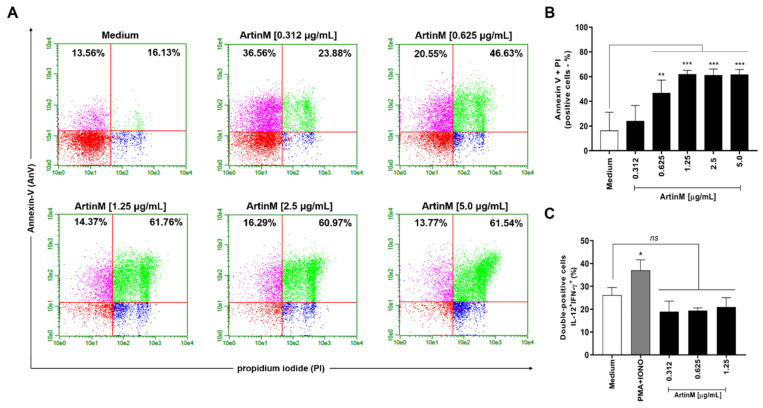
ArtinM increases the frequency of annexin V- and PI-positive B cells. B cells purified from a suspension of C57BL/6 mice spleen cells were seeded in 96-well microplates (5 × 10^5^ cells/well) and incubated for 24 h with ArtinM at several concentrations. Medium alone was used as a negative control (Medium). (**A**,**B**) B cells were labeled with annexin V FITC and propidium iodide (PI) to determine the frequency of apoptotic cells (annexin V + PI) by flow cytometry. (**C**) The frequency of IL-12+/IFN-γ+ double-positive cells was also determined by flow cytometry. A mixture of phorbol-12-myristate-13-acetate (PMA; 50 ng/mL) plus Ionomycin (IONO; 1 µM) was used as a positive control. Results are expressed as mean ± standard error of the mean (SEM), and three independent assays were performed. Significant differences compared to the medium are shown by *, *p* < 0.05; **, *p* < 0.01; and ***, *p* < 0.001. ns: not significant.

Considering our observation of reduced mitochondrial activity in murine B cells incubated for 24 h with ArtinM at high concentrations (Figure 1A), we examined the occurrence of apoptosis in ArtinM-stimulated B cells. Following 24 h incubation with 0.312–5.0 μg/mL ArtinM, we assayed B cells for annexin V (AnV) and propidium iodide (PI) markers. Compared with unstimulated B cells, B cells incubated with high concentrations of ArtinM showed an increase in AnV and PI (AnV+/PI+) double-positive cells in a dose-dependent manner (Figure 2A,B), indicating that ArtinM induces apoptosis in murine B cells. Then, the immunomodulatory activity of ArtinM at low concentrations in B cells was examined by verifying the relative frequency of IL-12- and IFN-γ-positive cells. Compared to unstimulated cells, stimulation with ArtinM at low concentrations (0.312–1.25 µg/mL) did not increase the frequency of IL-12+/IFN-γ+ double-positive B cells (Figure 2C). These data highlight the ArtinM preference for inducing apoptosis in murine B cells over immunomodulation for Th1 cytokine production.

### 2.3. ArtinM Interacts with Raji and Daudi Cells via Its CRD and Raji Cells Apoptosis Was More Pronounced in Response to ArtinM

**Figure 3:**
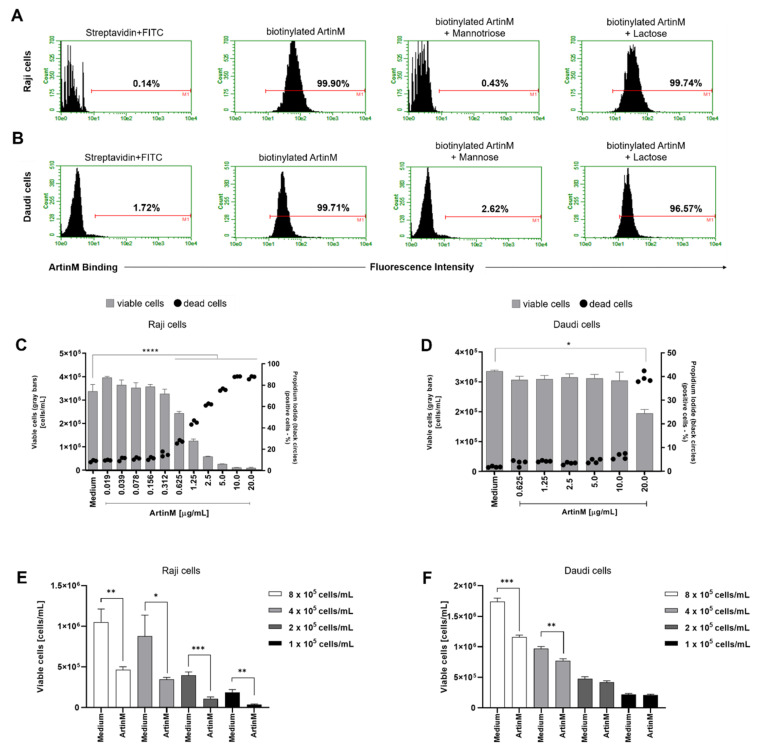
ArtinM binds Raji and Daudi cell surface through carbohydrate recognition, reducing Raji cells’ viability and increasing death. (**A**) Raji and (**B**) Daudi cells (1 × 10^6^ cells/mL) were fixed and incubated with biotinylated ArtinM (20 µg/mL), pre-treated or not with mannotriose (1 mM), mannose (20 mM) or lactose (20 mM). After washing, streptavidin-FITC (5 µg/mL) was added to the cells, which were analyzed by flow cytometry. Streptavidin-FITC alone was used as a negative control. Histograms represent the fluorescence intensity of cells for ArtinM detection; the positive range is indicated by the red line (M1). (**C**) Raji and (**D**) Daudi cells (1 × 10^5^ cells/mL) were distributed in 96-well microplates and incubated for 48 h at 37 °C with ArtinM at different concentrations. Medium alone was used as a negative control (Medium). The percentage of live and the frequency of dead (propidium iodide (PI)-positive) cells was determined by flow cytometry. The number of viable cells (grey bars) and the frequency of PI-positive cells (black circles) are shown in (**C**,**D**). (**E**,**F**) Raji and Daudi cells at different concentrations (1 to 8 × 10^5^ cells/mL) were distributed in 96-well microplates and incubated for 24 h at 37 °C with ArtinM (1.25 µg/mL for Raji cells and 20 µg/mL for Daudi cells). The concentration of viable cells was measured by flow cytometry using PI staining to determine the dead cells. The results are expressed as mean ± standard error of the mean (SEM); data are representative of three independent experiments performed in quadruplicate. Significant differences compared to the medium are shown by *, *p* < 0.05; **, *p* < 0.01; ***, *p* < 0.001; and **** *p* < 0.0001.

After verifying that ArtinM induces apoptosis of murine B cells, we investigated the cytotoxic effect of lectin on human B-cell lines sourced from NHL, i.e., Raji and Daudi cells. First, we verified the binding of ArtinM to the surface of both the cell lines (Figure 3A,B). In addition, we evaluated the inhibition of ArtinM binding by specific (mannose or mannotriose) and non-specific (lactose) sugars. As expected, only the specific sugars inhibited ArtinM binding to the cell surface. We then assayed propidium iodide incorporation by each cell line in response to ArtinM (0.02–20 µg/mL). The data obtained for Raji cells allowed us to verify the effect of lectin on cell growth and viability compared to the unstimulated cells (medium). The reduction in cell growth and viability in Raji cells was dose-dependent from 0.3 to 5 µg/mL ArtinM. In contrast, the growth and viability of Daudi cells were not affected by ArtinM at concentrations ranging from 0.625 µg/mL to 10 µg/mL. To evaluate the impact of cell concentration of Raji and Daudi cells in the induction of apoptosis by ArtinM, these cells were distributed in distinct concentrations (1 to 8 × 10^5^ cell/mL) and incubated with ArtinM for 24 h. The induction of Raji cells apoptosis triggered by ArtinM occurred in all cell concentrations tested (Figure 3E), whereas Daudi cells apoptosis was detected only at high cell concentration (Figure 3F). Taken together, these results demonstrated that Raji cells are more susceptible to apoptosis induced by ArtinM.

**Figure 4:**
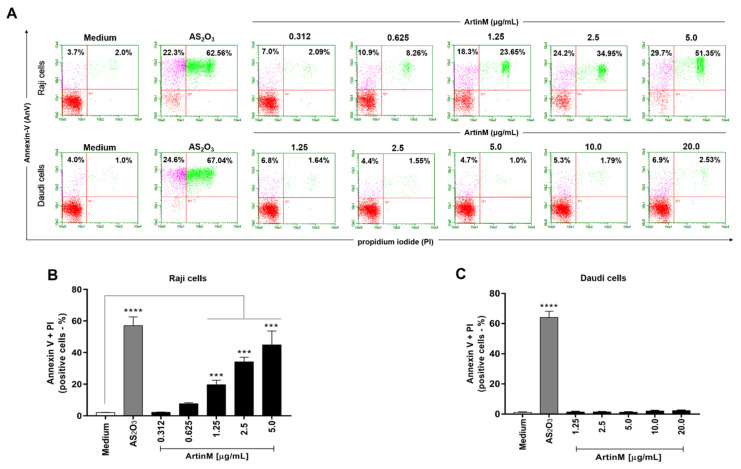
ArtinM induces apoptosis of Raji cells. Raji and Daudi cells (1 × 10^5^ cells/mL) were seeded in 96-well microplates and incubated for 48 h at 37 °C with 0.312 to 5.0 µg/mL and 1.25 to 20.0 µg/mL ArtinM, respectively. Arsenic trioxide (AS_2_O_3_) at concentrations of 12 µM (Raji cells) and 24 µM (Daudi cells) was used as a positive control for induction of cell death and medium alone was used as a negative control (Medium). The cells were incubated with annexin V-FITC (5.0 μg/mL) and propidium iodide (10.0 μg/mL), and the frequency of double-labeled (AnV+/PI+) Raji (**A**,**B**) and Daudi (**C**) cells was determined by flow cytometry. Values were expressed as mean ± standard error of the mean (SEM). Significant differences compared to the medium are shown by ***, *p* < 0.001 and ****, *p* < 0.0001.

The cytotoxic effect of ArtinM on B-NHL cell lines was also demonstrated by determining the frequency of Raji and Daudi cells for annexin V and propidium iodide positivity (AnV+/PI+) following 48 h stimulation with ArtinM. A dose-dependent increase in the frequency of double-positive Raji cells was observed (Figure 4A,B). In contrast, the percentage of annexin V- and PI-positive Daudi cells did not increase with ArtinM 48 h stimulation (Figure 4, panels A and C). These findings indicate that ArtinM exerts its cytotoxic effect on Raji cells via apoptosis.

### 2.4. Raji Cell Apoptosis Induced by ArtinM Was Reduced by a Pharmacological Inhibitor Specific to Caspases and Syk, Whereas the Relative Expression of Autophagy Markers Were Not Affected

**Figure 5:**
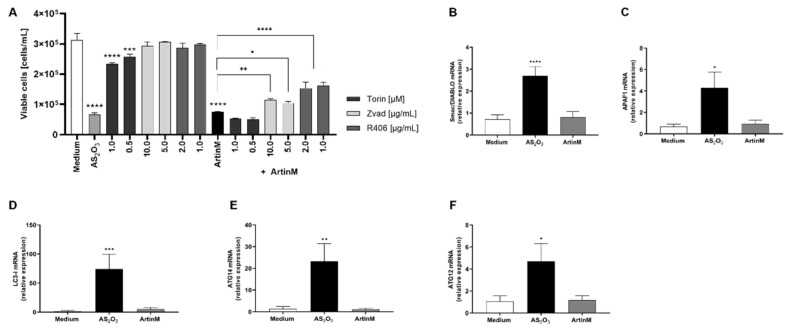
Apoptosis of ArtinM-stimulated Raji cells involves Syk and caspase molecules. Raji or Daudi cells (1 × 10^5^ cells/mL) were seeded in 96-well microplates and stimulated with ArtinM at concentrations of 1.25 µg/mL (Raji cells) or 20.0 µg/mL (Daudi cells) for 24 or 48 h at 37 °C. Arsenic trioxide (AS_2_O_3_), at concentrations of 12 µM (Raji cells) and 24 µM (Daudi cells), was used as a positive control of cell death induction; culture medium alone (Medium) was a negative control. (**A**) Pharmacological inhibitors Z-VAD (10 µg/mL), Torin-1 (1 µM), and R406 (1 µg/mL) were added to the culture, and after 210 min the ArtinM lectin (1.25 µg/mL) was incubated with Raji cells. After 24 h, the cells were harvested and stained with PI to determine the concentration of viable cells of Raji. (**B**–**F**) The relative expression of Smac/DIABLO (**B**), APAF1 (**C**), LC3-I (**D**), ATG14 (**E**), and ATG12 (**F**) was measured by qRT-PCR in ArtinM-stimulated Raji cells for 48 h. The Ct values of the target transcripts were normalized to the relative expression of β-actin as an endogenous control. The results are expressed as mean ± SEM, and the differences were considered significant at *p* < 0.05 (*), *p* < 0.001 (**), *p* < 0.001 (***), or *p* < 0.0001 (****).

The potent cytotoxic effects of ArtinM on Raji cells led us to investigate the involvement of caspase, mTOR, and Syk (spleen tyrosine kinase). Then, pharmacological inhibitors Z-VAD, Torin-1, and R406 were incubated with Raji cells with prior addition of ArtinM. After 24 h of incubation, the growth of Raji cells was measured by cell concentration using PI staining to determine only viable cells. The reduction of cell concentration of Raji cells caused by incubation with ArtinM was significantly altered when the cells were cultivated in the presence of Z-VAD or R406 prior to ArtinM stimulus (Figure 5A). Interestingly, 48 h stimulated Raji cells did not show increased apoptosis markers associated with the mitochondrial pathway (Apaf-1, and Smac/DIABLO) or autophagy (LC3-I, ATG14, and ATG12), as measured by the relative expression of their transcripts by qRT-PCR (Figure 5B–F). Considering the pronounced cytotoxic effect of ArtinM on Raji cells and the stable relative expression of all studied markers in response to ArtinM compared to unstimulated cells, we infer that the apoptosis of Raji cells induced by ArtinM depends partially on caspase and Syk.

### 2.5. TLR2 N-Glycans Are Involved in the Raji Cell Apoptosis Induced by ArtinM

**Figure 6:**
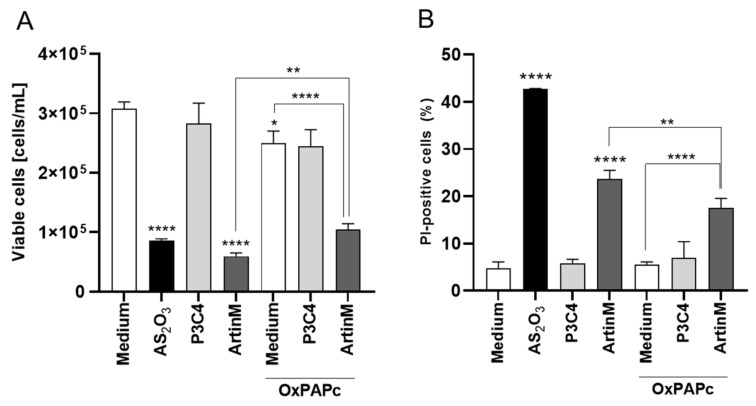
TLR-2 signaling blockaged by OxPAPC reduced the Raji cells apoptosis induced by ArtinM. Raji cells (1 × 10^5^ cells/mL), seeded in 96-well microplates, were incubated with OxPAPC (30 µg/mL) and ArtinM (1.25 µg/mL) at the same time. After 24 h, propidium iodide (10 μg/mL) was added to the cell culture and its incorporation was detected by flow cytometry, allowing the quantification of viable and non-viable cells. Raji cells were incubated with only medium, OxPAPC, or ArtinM as control. Additionally, Raji cells were also incubated with OxPAPC (30 µg/mL) and Pam3Csk4 (1.25 µg/mL) at the same time, and as control the cells were incubated with only Pam3Csk4 (1.25 µg/mL). Arsenic trioxide (AS_2_O_3_) at 12 µM was used as a positive control. Based on the total cell number and the frequency of PI+ cells (dead cells, black bars) measured by flow cytometry, the concentration of viable cells (**A**) and frequency of PI+ cells (**B**) were determined. The results are expressed as mean ± standard error of the mean (SEM) of three independent experiments carried out in triplicate. Significant differences compared to the medium or the ArtinM alone are shown by *, *p* < 0.05; ** *p* < 0.01; ****, *p* < 0.0001.

A previous study evidenced that Raji cell activation can be induced by TLR2 signaling [48], and the interaction between TLR2 N-glycans and ArtinM is well known [8,22]. To evaluate the involvement of TLR2 N-glycans in the Raji cells apoptosis induced by ArtinM, these cells were incubated with OxPAPC, which blocks the signaling of TLR2. OxPAPC, at a concentration of 30 μg/mL, and ArtinM (1.25 μg/mL), were added to a culture of Raji cells concomitantly, and after 24 h the concentration of viable cells was determined using PI staining by flow cytometry. Raji cells incubated with a pharmacological inhibitor of signaling of TLR2 had a significant increase in the concentration of viable cells in the presence of ArtinM when compared to those Raji cells incubated with only ArtinM (Figure 6). Interestingly, a TLR2 agonist (Pam3CsK4), at a concentration of 1.25 μg/mL, was incubated with Raji cells, and the reduction in the number of viable cells was not affected in the absence or not of OxPAPC (Figure 6). Thus, TLR2 N-glycans are targeted by ArtinM corroborating in the Raji cells apoptosis induced by lectin.

### 2.6. ArtinM-Induced Raji Cell Apoptosis Is Not Affected by Pharmacological Inhibitors of the p38 MAPK, JNK, ERK, PKC, and PTK Signalling Molecules

**Figure 7:**
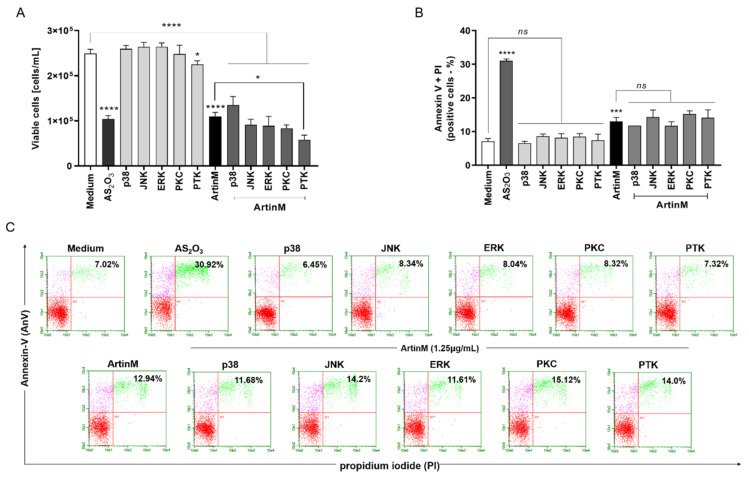
Pharmacological inhibition of the signaling molecules p38, JNK, ERK, PKC, or PTK does not affect ArtinM-induced apoptosis of Raji cells. Raji cells (1 × 10^5^ cells/mL) were seeded in 96-well microplates and pre-treated or not with pharmacological inhibitors (20µM), such as SB202190 (p38MAPK inhibitor), SP600125 (JNK inhibitor), PD98059 (ERK inhibitor), H-7 (PKC inhibitor) and genistein (PTK inhibitor). After 210 min, the cells were stimulated with ArtinM (1.25 µg/mL) for 24 h at 37 °C. Arsenic trioxide (AS_2_O_3_; 12 μM) was used as a positive control of cell death induction, and the medium alone was used as a negative control (Medium). (**A**) The number of viable cells (cells/mL) was determined as the difference between the total number of cells and PI+ cells (dead cells), detected by flow cytometry. The results are expressed as mean ± standard error of three independent experiments in triplicates. (**B**,**C**) The relative frequency of apoptotic cells was determined by annexin V-FITC (5.0 μg/mL) and PI (10.0 μg/mL) staining; double-positive cells (AnV+/PI+) were detected by flow cytometry analysis, and their relative frequency was represented as a percentage. Values with significant differences in relation to the medium or cells stimulated only with ArtinM are shown by *, *p* < 0.05; ***, *p* < 0.001; ****, *p* < 0.0001. ns: not significant.

Because the effect of ArtinM on apoptosis induction was confirmed in Raji cells (Figure 3 and Figure 4), we utilized the Raji cell line to search for specific signaling pathways that account for lectin-stimulated apoptosis. Next, we examined whether the apoptotic response to ArtinM was maintained in Raji cells pre-treated with pharmacological inhibitors of p38MAPK (SB202190), JNK (SP600125), ERK (PD98059), PKC (H-7), and PTK (genistein) signaling molecules. Raji cells were incubated with a pharmacological inhibitor (20 µM) for 210 min, followed by stimulation with ArtinM (1.25 µg/mL). After 24 h, flow cytometry (Figure 7) was performed; it was shown that the pharmacological inhibitors did not affect the number of viable cells (light gray bars, Figure 7A) or the frequency of apoptotic cells provided by the unstimulated cells (medium) (Figure 7B,C). In addition, the inhibitors, except genistein, did not affect the lower viable cell counts by ArtinM stimulus. The pre-treatment of Raji cells with Genistein, a PTK inhibitor, provided a cell count that was lower than that determined using ArtinM alone (dark grey bars, Figure 7A). We also analyzed the relative frequency of double-positive cells (AnV+/PI+) using flow cytometry. Cells pretreated with the inhibitors and ArtinM-stimulated cells contained a percentage of AnV+/PI+ cells that did not differ from those stimulated with ArtinM alone (Figure 7B,C). Although the PTK inhibitor reduced cell viability, it did not affect the apoptosis of Raji cells induced by ArtinM. Therefore, the effect of ArtinM on Raji cells was maintained even after selective pharmacological blocking of the signaling molecules p38, JNK, ERK, PKC, or PTK, indicating that none of the assayed signaling molecules accounted for ArtinM-induced apoptosis of Raji cells.

### 2.7. Phosphatase Activity of CD45 and Protein Kinases of the Src Family Account for the Cytotoxic Effect of ArtinM on Raji Cells

**Figure 8:**
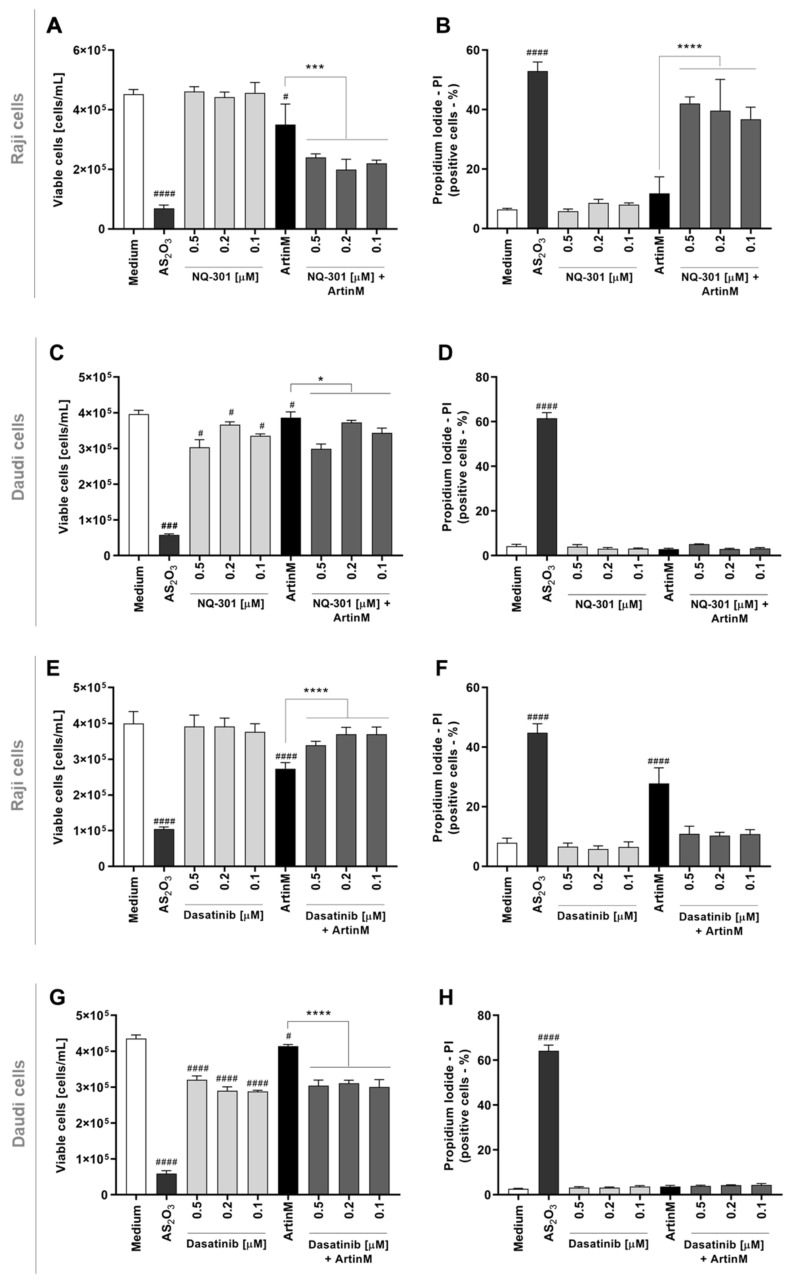
Pharmacological inhibitors of CD45 phosphatase activity and protein tyrosine kinases of the SCR family modify the cytotoxic effect of ArtinM in Raji cells. Raji (**A**,**B**,**E**,**F**) and Daudi (**C**,**D**,**G**,**H**) cells (1 × 10^5^ cells/mL), seeded in 96-well microplates, were pre-treated or not with the pharmacological inhibitors NQ-301 (**A**–**D**) or dasatinib (**E**–**H**) at different concentrations (0.5 to 0.1 µM) for 210 min, followed by stimulation with 1.25 µg/mL ArtinM (Raji cells) or 20 µg/mL ArtinM (Daudi cells). Arsenic trioxide (AS_2_O_3_) at 12 µM was used in Raji cells, and at 24 µM in Daudi cells, as a positive control. The unstimulated cells were used as a negative control. After 24 h, propidium iodide (10 μg/mL) was added to the cell culture and its incorporation was detected by flow cytometry, allowing the quantification of viable and non-viable cells. Based on the total cell number and the frequency of PI+ cells (dead cells, black bars), the quantification of viable cells (white bars) was determined. The results are expressed as mean ± standard error of the mean (SEM) of three independent experiments carried out in triplicates. Significant differences compared to the negative control (Medium) are shown by ^#^, *p* < 0.05; ^###^, *p* < 0.001; ^####^, *p* < 0.0001. Significant differences compared to the ArtinM alone are shown by *, *p* < 0.05; ***, *p* < 0.001; ****, *p* < 0.0001.

To further investigate the signaling pathways responsible for the cytotoxic effect of ArtinM on Raji cells, we evaluated whether inhibition of protein tyrosine kinases could modify apoptosis induced by ArtinM. We chose to study the effect of phosphatase activity of the CD45 and protein kinases of the Src family (Lyn, Fyn, and Blk) because of their known roles in regulating cell survival. Raji and Daudi cells were pre-treated with NQ-301, an inhibitor of dephosphorylation of the Y505 residue of the Lck molecule (lymphocyte-specific protein tyrosine kinase) of the CD45, or with Dasatinib, an inhibitor of the activity of Lyn, Fyn, and Blk kinases. The inhibitors were used at different concentrations (0.1, 0.2, and 0.5 μM) and, after 210 min, the cells were stimulated or not with ArtinM (1.25 µg/mL for Raji cells; 20.0 µg/mL for Daudi cells) for 24 h. We deduced the number of viable cells per mL by subtracting the number of PI+ cells detected by flow cytometry from the total number of cells. In Raji cells, treatment with either inhibitor (NQ-301 or dasatinib), not followed by stimulation with ArtinM, had no effect on cell viability (Figure 8A,E, light grey bars compared to the white bar). The same treatments modified the effect of ArtinM on the number of viable Raji cells, which was significantly reduced by NQ-301 (Figure 8A, dark gray bars compared to the black bar) and increased by dasatinib (Figure 8E, dark gray bars compared to the black bar). Conversely, the percentage of Raji cells that had incorporated PI, increased or decreased when stimulation with ArtinM was preceded by NQ-301 or dasatinib treatment, respectively (Figure 8B,F).

Only AS_2_O_3_ (positive control) promoted death in Daudi cells (Figure 8C,D,G,H). Upon stimulation with ArtinM or treatment with either inhibitor, the relative frequency of dead Daudi cells (PI+) remained as low as that observed in the negative control (medium) (Figure 8D,H). Treatment with either inhibitor, not followed by stimulation with ArtinM, significantly diminished the number of viable Daudi cells (Figure 8D). Increased viability and decreased apoptosis in Raji cells following pre-treatment with dasatinib in response to ArtinM (Figure 8C) indicated that Lyn, Fyn, and Blk kinases play a role in the cytotoxic effect of ArtinM on Raji cells. This result evidenced that dasatinib exerted its activity only on ArtinM-stimulated Raji cells and not on unstimulated Raji cells (Figure 8G). Our data indicated that the phosphatase activity of CD45 on Lck inhibits the cytotoxic effect of ArtinM in Raji cells. In contrast, protein kinases of the Src family favor cell death triggered by ArtinM.

**Figure 9:**
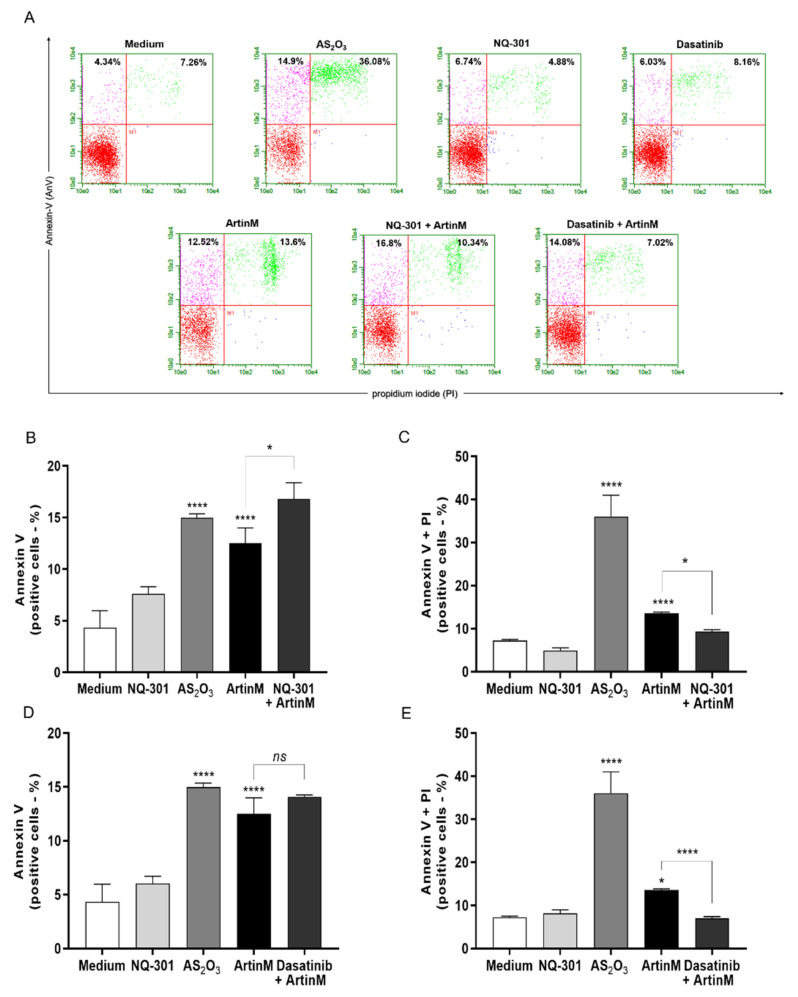
NQ-301 and dasatinib differently affect apoptosis of ArtinM-stimulated Raji cells. Raji cells (1 × 10^5^ cells/mL) were seeded in 96-well microplates and pre-treated or not with the pharmacological inhibitors NQ-301 (**A**–**C**) or dasatinib (**A**–**E**) at a concentration of 0.2 µM for 210 min. The cells were stimulated with ArtinM (1.25 µg/mL). Arsenic trioxide (AS_2_O_3_; 12 µM) was used as a positive control of cell death, and medium alone as a negative control. After 24 h stimulation, the cells were incubated with AnV-FITC (5.0 μg/mL) and PI (10.0 μg/mL) and analyzed by flow cytometry to determine the relative frequency (%) of Raji cells labeled only with annexin V (AnV+) (**B**,**D**) or double-labeled annexin V-FITC plus PI (AnV+/PI+) (**C**,**E**). Significant differences compared to the medium or the isolated stimulus with ArtinM are shown by *, *p* < 0.05; ****, *p* < 0.0001.

To validate that NQ-301 and dasatinib regulate cell death triggered by ArtinM, we used Raji cells pretreated with these inhibitors. We stimulated Raji cells with ArtinM and analyzed them for apoptosis markers to determine the frequency of AnV+ (early apoptosis marker) and AnV+/PI+ (late apoptosis marker) cells. We found that both pre-treatments with NQ-301 and dasatinib led to a reduced frequency of AnV+/PI+ Raji cells in response to ArtinM, compared to cells stimulated with ArtinM alone (Figure 9C,E). However, AnV+ Raji cell frequency in response to ArtinM was augmented by NQ-301 (Figure 9B) and was not altered by dasatinib pre-treatment (Figure 9D). These data indicate the negative regulation by CD45 in ArtinM-stimulated apoptosis of Raji cells, while the mediation of the cytotoxic effect of ArtinM by protein kinases of the Src family can be seen in Raji cells.

**Figure 10:**
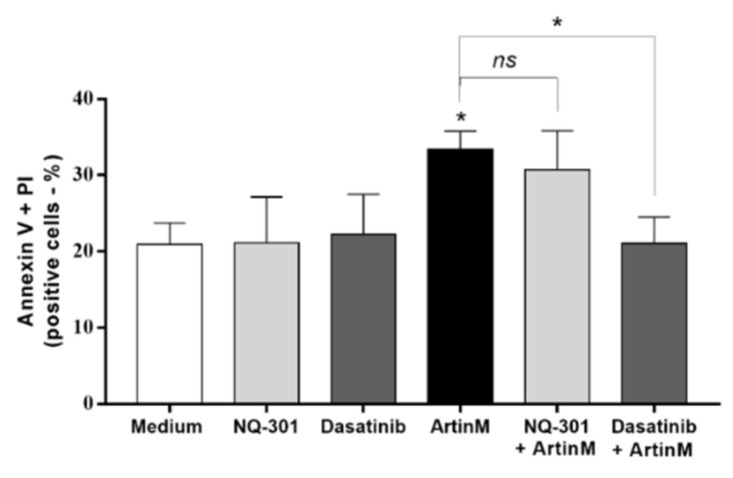
Dasatinib inhibits apoptosis of murine B cells induced by ArtinM. Murine B cells (5 × 10^5^ cells/mL) seeded in 96-well microplates were pre-treated or not with 0.1 µM NQ-301 or dasatinib for 210 min. The cells were stimulated or not with ArtinM (0.625 µg/mL). The medium was used as a negative control. After 24 h, the cells were labeled with annexin V-FITC (5.0 μg/mL) and PI (10.0 μg/mL), and the frequency (%) of AnV+/PI+ B cells was determined by flow cytometry. Significant differences compared to the medium or stimulus with ArtinM are shown by *, *p* < 0.05.

To evaluate the effect of NQ-301 and dasatinib on ArtinM-stimulated apoptosis in normal B cells, they were purified from a suspension of mouse spleen cells. We treated the B cells with NQ-301 (0.1 μM) or dasatinib (0.1 μM), followed by stimulation with ArtinM (1.25 μg/mL). Pre-treatments only did not induce an apoptotic response in B cells, and ArtinM stimulus increased the frequency of AnV+/PI+ cells as expected. We detected a higher frequency of AnV+/PI+ cells in ArtinM-stimulated B cells pretreated with NQ-301, but not in dasatinib-treated cells (Figure 10). Therefore, Src family protein kinases, and not CD45, participate in ArtinM-induced apoptosis of murine B cells.

### 2.8. Quantitative Proteomics Analysis to Identify Possible Targets Whose Expression Is Regulated by the Cytotoxic Effect of Artinm

A quantitative proteomics approach was used to identify cell signaling molecules possibly involved in the regulation of the survival of Raji and Daudi cells stimulated with ArtinM. We searched for proteins whose differential expression in NHL B-cell lines was associated with the cytotoxic effect of ArtinM. A total of 2091 proteins were identified, of which 921 were quantified for both cell lines under different experimental conditions (Table 1A). Table 1 lists the top 10 upregulated and downregulated proteins in both Raji and Daudi cells following stimulation with ArtinM. In Raji cells, we identified upregulated proteins (Table 1A), including HLA-B (P01889) and HLA-DRA (P01903), involved in B-cell death. Eukaryotic translation initiation factors, such as eIF3-E (P60228) and eIF3-M (Q7L2H7), were also upregulated. Downregulated proteins, including those associated with the regulation of cell proliferation and survival, such as MIF (P14174) and PDLIM1 (O00151), were identified in ArtinM-stimulated Raji cells (Table 1B). In Daudi cells (Table 1C), positive regulators of cell cycle progression, namely, ANP32B (Q92688), GSPT1 (P15170), and SUB1 (P53999) were upregulated after ArtinM stimulus. In addition, ArtinM-stimulation of Daudi cells up-regulated proteins was involved in the transcriptional regulation of histones H1-2 (P16403), H1-4 (P10412), and H2AC11 (P0C0S8). In contrast, essential proteins that inhibit cell proliferation and induce apoptosis, such as NAT10 (Q9H0A0), PSMB2 (P49721), and RAB7A (P51149), were downregulated in the ArtinM-stimulated Daudi cells (Table 1D).

## 3. Discussion

Our group previously demonstrated, in vitro and in vivo, the role of ArtinM in modulating innate [8,22,24,49,50] and adaptive [23,51] immune responses. We have determined that the interaction of ArtinM with N-glycans linked to glycoproteins on the cell surface, such as TLR2/CD14 and CD3εγ, provides the primary mechanism for triggering the production of cytokines responsible for the immunomodulation induced by ArtinM [8,22,52,53]. However, the recognition of TLR2/CD14 N-glycans does not account for the ArtinM activity that induces murine B cells to produce IL-17 and IL-12p40 [26]. Notably, the subcutaneous administration of ArtinM to naive BALB/c mice, besides affecting immune cells, increases the number of splenic B cells [54], suggesting that B cells contribute to the immunomodulatory activity of ArtinM in vivo. To explore this premise, we investigated the effect of ArtinM on isolated murine B cells and found that ArtinM is not directly responsible for B cell proliferation in vivo. Instead, lectin promotes murine B cell apoptosis. This cytotoxic effect of ArtinM was also detected in Raji cells, a human B-cell line sourced from NHL. The phosphatase activity of CD45 and protein kinases of Syk and Src family are major molecules involved in the cytotoxic effects of ArtinM on Raji cells.

The general characterization of the biological effects of lectins on distinct cell types frequently includes measurement of mitochondrial activity by MTT assay, which allows screening if the explored lectins induce cell proliferation (high mitochondrial activity, likely linked to a cell activation process) or apoptosis (low mitochondrial activity, probably accounting for cytotoxic effects) [55,56,57,58,59,60]. We found a dose-dependent decrease in mitochondrial activity in B cells that were stimulated for 24 h with ArtinM at the higher concentrations tested, indicating that the lectin could trigger B cell apoptosis. Indeed, it was confirmed that ArtinM diminished the number of viable cells and increased the frequency of double-positive B cells for AnV and PI, which are flow cytometric criteria used to identify cell apoptosis. The association between reduced mitochondrial activity and cytotoxic effects of ArtinM has been reported when stimulating a few cell types, such as murine CD4^+^ and CD8^+^ T cells [23], NB4 human myeloid leukemia cells [27], and Jurkat human leukemic T cells [23]. Beyond the decrease in mitochondrial activity determined by 24 h stimulation with ArtinM at high concentrations, the MTT assay also revealed a small augmentation of mitochondrial activity in B cells stimulated for 48 h with ArtinM at low concentrations compared with the negative control. This could imply the occurrence of B cell proliferation; however, we did not find a concomitant augmentation of thymidine incorporation or IL-2 production by B cells stimulated by ArtinM. Few studies have reported on the effects of lectins on B cell proliferation or apoptosis [4,61]. Interestingly, jacalin, the Galβ1-3Gal-NAc-binding lectin, is also sourced from *A. heterophyllus* seeds and induces B-cell apoptosis, which results from lectin interaction with CD45 O-glycans [62].

We initially aimed to determine the contribution of B cells to the Th1 immune response, which was induced after ArtinM administration to mice experimentally infected with an intracellular pathogen to which they were susceptible. We found that isolated murine B cells stimulated in vitro with ArtinM, even when used at concentrations with low cytotoxic effects, did not respond by producing IL-12 and IFN-γ. Instead, ArtinM-stimulated B cells become apoptotic rather than proinflammatory. Because of the high cytotoxic effect exerted by ArtinM on B cells, we aimed to investigate lectin activity in human B-cell lines derived from Burkitt lymphoma, a subtype of NHL [63]. We first confirmed that ArtinM binds to Raji and Daudi cell surface, in a manner dependent on carbohydrate recognition. This phenomenon also occurs with other lectins from different sources having specificity for distinct glycoligands, such as SAL (*Silurus asotus*), which has specificity for α-galactoside ligands [64,65], CGL (*Crenomytilus grayanus*) for Galα1-4Galβ1-4GlcNAc ligands [66], MytiLec (*Mytilus galloprovincialis*) for α-GalNAc ligands [67], and OLL (*Osmerus lanceolatus*) for globotriaosylceramide ligands [68]. By recognizing glycoligands on the Raji and Daudi cell surface, the reported lectins reduced cell viability as ArtinM did. We found that the cytotoxic effect of ArtinM was pronounced in Raji cells, which diminished their growth and augmented their frequency of apoptosis. Diminished Raji cell viability was previously described to be induced by a few lectins, such as jacalin (*Artocarpus heterophyllus*) [69], SAL (*Silurus asotus*) [68], and ALL (*Artocarpus lingnanensis*) [70].

Our present work showed that ArtinM did not induce Raji cell autophagy, as demonstrated by the reduced levels of relative expression of specific transcripts for the autophagy pathway. In contrast, we previously reported that ArtinM induces autophagy in NB4 cells without caspase involvement [27]. The lectins Abrin (*Abrus precatorius*) and CvL (*Cliona varians*) induce apoptosis in human acute lymphoblastic leukemia (CCRF-CEM) and human erythroleukemia (K562) cell lines, respectively, in a manner that is independent of caspase activity [71,72]. Our study showed that ArtinM-induced apoptosis of Raji cells was reduced by caspases inhibitor (Z-VAD), and further studies should be performed to clarify which caspase is involved with the Raji cells apoptosis induced by ArtinM.

To provoke a cell response, extracellular signals activate several signaling pathways to regulate proliferation, survival, cell cycle arrest, and cell death [73]. In this sense, Raji cells apoptosis induced by the lectin ALL, an N-acetyl-D-galactosamine ligand, was reported as being mediated by the p38MAPK signaling pathway [70]. Another study showed that MytiLec, a Galα1-4Galβ1-4Gl-specific lectin, recognizes Ramos cells (BL lineage) and induces apoptosis via activation of the MEK/ERK and JNK/p38 MAPK pathways [67]. Various signaling pathways were assessed as being possibly involved in ArtinM-induced Raji cell apoptosis. The pathways were approached by pharmacological inhibition of the protein kinases p38MAPK, JNK, ERK, PKC, and PTK. The role of either molecule was evaluated by measuring the cell growth and frequency of double-positive cells for AnV and PI in B cell cultures after incubation with inhibitors followed by the ArtinM stimulus. The participation of PTK was detected in the ArtinM-stimulated reduction of Raji cell growth, and PTK may be an important negative regulator of ArtinM activity in Raji cells. Our group reported the involvement of PTK in the apoptosis of Jurkat cells (T-lymphocyte lines from leukemic cells) induced by ArtinM, which acts as a positive regulator of Jurkat cell activation in response to ArtinM, resulting in the apoptosis of Jurkat cells [23].

To address this hypothesis, the present study also examined the B cell antigen receptor (BCR) signaling cascade as a possible major mediator of Raji cell apoptosis induced by ArtinM. Of the components involved in BCR signaling, the protein kinases of the Src family and CD45 participate strictly in pro-survival signal propagation [61], acting as positive and negative regulators, respectively, of the signaling cascade from BCR [74]. Several studies have reported that CD45 expressed on B cells is targeted by lectins such as PNA (*Arachis hypogaea*) [75], jacalin [62], and Galectin-9 [76]. The recognition of CD45 on B cell surface by Galectin-9 suppresses B cell activation and cell proliferation through Lyn activation, which mediates inhibitory signaling pathways associated with the CD22 co-receptor and SHP-1 tyrosine phosphatase [76]. We investigated the influence of CD45 regulatory activity on the growth and viability of ArtinM-stimulated Raji cells. Using the pharmacological inhibitor NQ301, we blocked the dephosphorylation of the Y505 residue promoted by Lck molecule through CD45 phosphatase activity and verified the enhancement of ArtinM-induced Raji cell apoptosis, suggesting that CD45 phosphatase activity acts as a negative regulator of the lectin effect. It is known that during BCR engagement in chronic lymphoid leukemia (CLL) cells, CD79α phosphorylation is induced by Lck, which induces activation of PI3K/Akt, NF-κB, and MAPK, promoting the survival of stimulated CLL cells [77]. In addition, Src family protein kinases (SFKs), such as Lyn, Fyn, and Blk, are required for several B-cell functions, including differentiation, proliferation, and survival [39]. SFKs are also regulated by CD45 phosphatase activity [78], and their role in Raji cell apoptosis was verified using the pharmacological inhibitor dasatinib. Inhibition of Src family kinases blocked the ArtinM-stimulated reduction of Raji cell growth and viability. These data indicate that the kinases of the Src family and CD45 phosphatase activity regulate the cytotoxic effect of ArtinM on Raji cells.

The cytotoxic effect of ArtinM was more evident in Raji cells than in Daudi cells. This difference allowed us to establish convenient comparisons of the biological facts determined by ArtinM stimulation in the two cell lines. It has been considered, in the proteomic approach, to discriminate discrepant and valuable molecules upregulated or downregulated in each cell line undergoing the ArtinM effects. Our data demonstrated the upregulation of major histocompatibility complex (MHC) class II molecules in ArtinM-stimulated Raji cells. MHC-II molecules, including HLA-DR, HLA-DQ, and HLA-DP, were already reported to participate in the process of inducing cell death [79,80]. Remarkably, the ability of HLA-DR to trigger apoptosis in healthy and neoplastic B-lymphocytes has been reported [81,82]. Consistently, Bertho et al. demonstrated the induction of Raji cell apoptosis via HLA-DR signaling [83]. Apoptosis of B cells triggered by HLA-DR occurs in a caspase-independent manner [84]. Proteomic analysis of ArtinM-stimulated Daudi cells revealed that histones and acidic nuclear phosphoprotein 32 family member B (ANP32B) were the most abundantly detected proteins. Interestingly, ANP32B downregulation exerts anti-apoptotic effects, whereas ANP32B acts as a transcriptional regulator of genes involved in cell cycle progression [85,86]. In human leukemic cell lines, ANP32B downregulation enhances caspase-3 activation, leading to cell apoptosis [87]. ANP32B downregulation has been shown to exert anti-apoptotic effects on hepatocellular carcinoma [88] and breast cancer [89] cells. The ANP32B protein acts as a histone chaperone; its direct binding to histones regulates gene transcription, which affects acetylation and phosphorylation inhibition [90,91,92]. Taken together, our results suggest that MHC-II molecules are involved in the apoptosis of ArtinM-induced Raji cells, which may explain the absence of DNA fragmentation. However, the regulation triggered by ANP32B in Daudi cells could inhibit the cytotoxic effect of ArtinM in Daudi cells.

The aberrant glycosylation is usually reported in cancer cells and the profile of glycans can be used as a hallmark of cancer [93], and plant lectins are tools to identify the alteration in glycosylation [94]. In addition, the biological effects of the aberrant glycosylation are reflected in the signal transduction and cellular communication that favor the tumor cell invasion, tumor angiogenesis, and metastasis formation [2]. These findings demonstrated the importance of the glycobiology as a promising field to prospect potential biomarkers specific to cancer cells and also to identify glycans as target to the development of new therapeutic approaches against cancer. In this context, the current study evidenced that the glycans targeted by ArtinM had high relevance in the control of the cellular expansion of NHL cell lineage.

We conclude that the cytotoxic effect of ArtinM on murine B cells is largely prominent and reproducible in Raji cells, an NHL cell line. Apoptosis induced by ArtinM in B cells derived from NHL is strongly regulated by CD45 phosphatase activity, Src family kinases, and Syk.

## 4. Materials and Methods

### 4.1. Animals

Male C57BL/6 mice at 6–8 weeks of age were used in this study. The mice were acquired from the animal facility of Ribeirão Preto Medical School at the University of São Paulo, Ribeirão Preto, São Paulo, Brazil. The animals were bred and housed under optimized hygienic conditions at the animal facility of the Department of Cell and Molecular Biology and Pathogenic Bioagents at the Ribeirão Preto Medical School. All experimental procedures were performed in accordance with the local animal ethical committee of Ribeirão Preto Medical School of the University of São Paulo (protocol number: 061/2019).

### 4.2. Purification of ArtinM Lectin

ArtinM lectin was purified from the saline extract of *Artocarpus heterophyllus* (jackfruit) seeds using affinity chromatography with D-mannose columns coupled to Sepharose^®^ (Pierce Chemical Company, Dallas, TX, USA), according to previously described protocols by Da Silva et al. [19].

### 4.3. Murine B Cells Isolation

Suspensions of spleen cells obtained from mice were prepared as reported by Oliveira-Brito et al. [26] using the B cell enrichment kit (Pan B Cell Isolation Kit, Miltenyi Biotec, Auburn, CA, USA), according to the manufacturer’s instructions. The negatively selected cells were stained with PE Rat Anti-Mouse CD19 (BD Pharmingen™) antibodies and analyzed by flow cytometry (Guava^®^ easyCyte, Millipore, Burlington, MA, USA). The isolation of B cells reached about 96% of positive cells for CD19 marker.

### 4.4. Burkitt’s Lymphoma Cell Lines

Raji and Daudi cell lines derived from Burkitt’s lymphoma were routinely grown in advanced Roswell Park Memorial Institute 1640 medium (Gibco^®^, Life Technologies, Carlsbad, CA, USA) supplemented with 10% fetal bovine serum, 1% streptomycin/penicillin, 1 mM sodium pyruvate, and 2 mM L-glutamine, in a humidified 5% CO_2_ atmosphere at 37 °C. Raji and Daudi cells were gifts from Dra. Fabíola Traina (Department of Medical Images, Hematology and Clinical Oncology, Hospital of the Ribeirão Preto Medical School, University of São Paulo, Ribeirão Preto, Sao Paulo, Brazil).

### 4.5. ArtinM Binding on Raji and Daudi Cell Surface

Raji and Daudi cells (1 × 10^6^ cells/mL) were fixed with phosphate-buffered saline (PBS)/3% formaldehyde for 20 min on ice, and washed with PBS or 1% glycine. The cells were then washed twice with PBS and incubated with biotinylated ArtinM (20 µg/mL) that was pre-incubated with or without mannotriose (1 mM), mannose (20 mM), lactose (20 mM), or medium alone for 40 min. The cells were then washed with PBS and incubated with streptavidin–fluorescein isothiocyanate (FITC; 5 µg/mL; Invitrogen) for 40 min. The cells were washed with PBS, and biotinylated ArtinM was detected by flow cytometry (Guava^®^ easyCyte, Millipore, Burlington, MA, USA). Raji and Daudi cells were incubated with streptavidin-FITC alone (5 µg/mL) in the absence of lectin and were used as a negative control. The percentage of positive cells was plotted as a histogram.

### 4.6. Determination of Mitochondrial Activity by 3-(4,5-Dimethylthiazol-2-yl)-2,5-diphenyltetrazolium Bromide (MTT)

Purified murine B cells (5 × 10^5^ cells/well) were stimulated with ArtinM (0.312–5.0 µg/mL) for 24 or 48 h at 37 °C. PMA (50 ng/mL) plus ionomycin (1 µM) was used as a positive control for cell activation, and cells incubated with medium alone were used as negative controls. The mitochondrial activity of the cells was determined after the reduction of MTT (Sigma-Aldrich) to produce formazan crystals [95]. The procedure was performed as described by da Silva et al. [51]. Mitochondrial activity was expressed as a percentage after comparison with the absorbance of unstimulated B cells (medium).

### 4.7. Cell Proliferation Assay and Measurement of IL-2 Levels

Purified murine B cells (5 × 10^5^ cells/well) were stimulated with ArtinM (0.312–5.0 µg/mL), PMA (50 ng/mL) plus ionomycin (1 µM), or the medium alone. After 48 h of incubation at 37 °C, [^3^H]-thymidine ([^3^H]-TdR; Amersham Bioscience, Boston, MA, USA) at 0.5 µCi/well was added and the proliferative rate was measured by [^3^H]-TdR incorporation. The detection of [^3^H]-TdR incorporation was measured using a scintillator, and the results were expressed as counts per minute (CPM) and the stimulation index of cell proliferation. After 48 h of incubation, murine B cells were centrifuged (300× *g*, 10 min at room temperature) and the supernatants were collected to measure the levels of IL-2 by ELISA using the OptEIA™ kit (BD Biosciences, San Jose, CA, USA), according to the manufacturer’s instructions.

### 4.8. Intracellular Staining of IL-12 and IFN-γ

Purified murine B cells (5 × 10^5^ cells/well) were stimulated with ArtinM (0.312–5.0 µg/mL), PMA (50 ng/mL) plus ionomycin (1 µM), or culture medium alone. After 12 h of incubation at 37 °C, the protein transport inhibitor was added (BD GolgiStopTM; 1 µL every 1.5 mL of the culture), and after 12 h the cells were washed and fixed/permeabilized with BD Cytofix/CytopermTM Plus (20 min at 4 °C). The cells were washed with wash buffer (BD Perm/WashTM buffer) and resuspended in Perm/WashTM buffer with anti-IFN-γ PE (BD Pharmingen™) and anti-IL-12 FITC (BD PharmingenTM) antibodies. After 40 min of incubation at 4 °C, the cells were washed and resuspended in Perm/WashTM buffer, and the frequency of positive B cells for IL-12 and IFN-γ (IL-12+/IFN-γ+) was measured using flow cytometry (Guava EasyCyte™ Mini System).

### 4.9. Quantification of Positive Cells for Annexin V (AnV) and/or Propidium Iodide (PI)

Purified murine B cells (5 × 10^5^ cells/well), Raji cells, or Daudi cells (1 × 10^6^ cells/mL) were stimulated with different concentrations of ArtinM (0.019–20.0 µg/mL), PMA (50 ng/mL) plus Ionomycin (1 µM), Arsenic trioxide (AS_2_O_3_; 12 µM or 24 µM) or culture medium alone at 37 °C. After 24 or 48 h, as specified in the figure legends, the cells were stained with annexin V-FITC (5 µg/mL; 40 min at 37 °C), and propidium iodide (10 µg/mL) was added at the time of analysis. The frequency of positive cells for AnV binding and/or PI incorporation was quantified by flow cytometry (Guava EasyCyte™ Mini System).

### 4.10. qRT-PCR for Determining Apoptosis Marker Expression

Raji cells (1 × 10^6^ cells/mL) were stimulated with ArtinM (1.25 µg/mL), Arsenic trioxide (AS_2_O_3_; 12 µM), or medium alone for 24 h at 37 °C. Total RNA was extracted from Raji cells using TRIzol according to the manufacturer’s instructions, and the RNA was converted into cDNA using the *iScript*™ *cDNA synthesis kit (Bio-Rad)*. qRT-PCR was performed in a final volume of 10 μL using EvaGreen (Bio-Rad) and the quantification was done by using CFX96 (Bio-Rad) equipment. The cycling conditions were as follows: initial denaturation at 95 °C for 30 s, followed by 40 cycles of denaturation at 95 °C for 5 s, and annealing/extension at 60 °C for 5 s. The relative expression of the transcripts was quantified using the ΔΔCt method and normalized to β-actin expression. The specificity of amplification was determined using melting curve analysis. The following PCR primers were used: β-actin (F-GTTGTCGACGACGAGCG, R-GCACAGAGCCTCGCCTT), Apaf-1 (F-CCTCTCATTTGCTGATGTCG, R-TCACTGCAGATTTTCACCAGA), Smac/Diablo (F-AGCTGAATGTGATTCCTGGC, R-GAAGCTGGAAACCACTTGGA), LC3-I (F-TATCACCGGGATTTTGGTTG, R-GAGAAGACCTTCAAGCAGCG), ATG14 (F-TCTTGCTTGCTCTTAAGTCGG, R-GAGCGGCGATTTCGTCTACT), and ATG-12 (F-CCATCACTGCCAAAACACTC, R-TTGTGGCCTCAGAACAGTTG).

### 4.11. Inhibition Assay of Signaling Molecules in Raji and Daudi Cells Stimulated with ArtinM

Raji and Daudi cells (1 × 10^6^ cells/mL) were incubated, separately, with the following pharmacological inhibitors: SB202190 (p38MAPK inhibitor), SP600125 (JNK inhibitor), PD98059 (p42/44MAPK inhibitor), H-7 (PKC inhibitor), Genistein (PTK inhibitor), Z-VAD (caspases inhibitor), Torin-1 (mTOR inhibitor), R406 (Syk inhibitor), NQ-301 (inhibitor of CD45 phosphatase activity on Lck) and Dasatinib (inhibitor of Lyn, Fyn, and Blk) for 210 min at 37 °C. The cells were subsequently stimulated with ArtinM at concentrations of 1.25 µg/mL (Raji cells), and 20.0 µg/mL (Daudi cells), for 24 or 48 h at 37 °C. Arsenic trioxide (AS_2_O_3_), at concentrations of 12 µM (Raji cells) and 24 µM (Daudi cells), was used as a positive control, and the medium alone was used as a negative control. The frequency of positive cells for AnV binding and/or PI incorporation was quantified by flow cytometry (Guava EasyCyte™ Mini System), as described in Section 4.9.

### 4.12. In-Solution Trypsin Digestion

In-solution trypsin digestion was performed according to the protocol described by Pessotti et al. [96]. Briefly, a solution of 6 M guanidine hydrochloride (GuHCl) was added to 100 μg of protein under each condition to a make up a final concentration of 3 M GuHCl, followed by the addition of 5 mM dithiothreitol (DTT) (final concentration). The mixture was incubated at 65 °C for 60 min. Iodoacetamide (IAA) was then added to a final concentration of 15 mM, and the samples were incubated for 60 min at room temperature in the dark. To quench the excess IAA, DTT was added to a final concentration of 15 mM. Clean-up of the samples was performed by the addition of ice-cold acetone (8 volumes) and methanol (1 volume), followed by incubation of the samples for 3 h at −80 °C. After centrifugation at 14,000× *g* for 10 min, protein pellets were washed twice with one volume of ice-cold methanol and then solubilized with NaOH solution (final concentration of 2.5 mM), followed by the addition of 50 mM HEPES buffer, pH 7.5, to a final volume of 100 mL. Trypsin (Proteomics grade; Sigma, USA) was added at 1:100 (enzyme/substrate) ratio and protein samples were incubated at 37 °C for 18 h. Tryptic peptides were desalted using C18 StageTips [97], dried in a SpeedVac, and re-dissolved in 50 mL of 0.1% formic acid prior to nanoflow liquid chromatography/tandem mass spectrometry (LC-MS/MS) analysis.

### 4.13. Mass Spectrometry

LC-MS/MS analyses were performed on a Synapt G2 HDMS mass spectrometer (Waters) coupled to the chromatographic system nanoAcquity UPLC (Waters). Digested peptide samples (7 µg) were loaded onto a trap column (Acquity UPLC M-Class Symmetry C18 Trap Column, 100 Å, 5 μm, 300 μm × 25 mm, Waters) at 8 μL/min of loading solution (5% acetonitrile and 0.1% formic acid in water) for 5 min. Then, the mixture of trapped peptides was eluted in an analytical column (Acquity UPLC M-Class HSS T3 Column, 1.8 μm, 300 μm × 150 mm, Waters) with a gradient of 5–35% of phase B (0.1% formic acid in acetonitrile, phase A: 0.1% formic acid in water), over 60 min at a flow rate of 3 µL/min. MS data were acquired in data-independent acquisition (DIA) mode UDMS^E^ using ion mobility separation [98] in the m/z range of 50–2000 and in the resolution mode. Peptide ions were fragmented by collision-induced dissociation (CID), in which collision energies were alternated between low (4 eV) and high energy (ramped from 17 to 60 eV) for precursor and fragment ions, respectively, using scan times of 1.0 s. The ESI source was operated in the positive mode with a capillary voltage of 2.7 kV, block temperature of 100 °C, and cone voltage of 40 V. The column temperature was set to 55 °C. For lock mass correction, a [Glu1]-Fibrinopeptide B solution (500 fmol/mL in 50% methanol, 0.1% formic acid; Peptide 2.0) was infused through the reference sprayer at 2 μL/min and sampled every 60 s for external calibration [99].

### 4.14. Quantitative Proteomics

Label-free quantification (LFQ) was performed using Progenesis QI for proteomics (Nonlinear Dynamics), as previously reported [100,101]. Briefly, the raw files were loaded into the software, and the peaks were aligned based on the precursor ion retention time of the reference run, which was automatically selected. Default peak-picking parameters were applied. The MS data were processed by the Apex3D module using a low-energy threshold of 750 counts and a high-energy threshold of 50 counts. The MS/MS spectra were exported as a mgf file for protein identification in PEAKS Studio 7.5 (Bioinformatics Solution, Inc., Waterloo, ON, Canada). A search was performed against *Homo sapiens* sequences in the UniprotKB/Swissprot database (www.uniprot.org; reviewed: 26,577 sequences; downloaded on 10 May 2021). Search parameters were set as follows: mass tolerance of 10 ppm for precursor ions and 0.025 Da for fragment ions, up to two missed cleavage sites allowed for trypsin digestion, maximum false discovery rate (FDR) of 1% at the peptide level, and a minimum of two peptides per protein. Carbamidomethylation of cysteines was selected as a fixed modification, and N-terminal acetylation, methionine oxidation, and asparagine/glutamine deamidation were selected as variable modifications. The identification results were then reimported to the Progenesis QI for proteomics as a .pepXml file. Proteins were quantified as the sum of all unique (non-conflicting) normalized peptide ion abundances corresponding to the protein. Mass spectrometry proteomics data were deposited in the ProteomeXchange Consortium via the PRIDE [1] partner repository with the dataset identifier PXD029399 (Reviewer account details: Username: reviewer_pxd029399@ebi.ac.uk, Password: 4TrQpmLp).

### 4.15. Statistical Analysis

The results are presented as the mean ± standard error of the mean (SEM). All data were analyzed using Prism 7.0 (GraphPad Software, La Jolla, CA, USA). All statistical determinations for normality were analyzed using the Kolmogorov–Smirnov test with the Dallal–Wilkinson–Lillie test for p-values, and when the distribution could not be assumed to be normal, the Kruskal–Wallis test was used. Statistical determinations of the differences in means between groups were performed using the Kruskal–Wallis test followed by Dunn’s multiple comparison test. The experiments were performed in triplicates or quadruplicates, and three independent assays were performed. Differences with *p* < 0.05 (*); *p* < 0.01 (**); *p* < 0.001 (***); *p* < 0.0001 (****) were considered statistically significant.

## Figures and Tables

**Table 1:** The top 10 upregulated (**A**,**C**) and downregulated (**B**,**D**) proteins in Raji and Daudi cells after treatment with ArtinM.

(**A**)			
**Gene Name**	**Protein Name**	**Accession**	**Fold-Change**
HLA-B	HLA class I histocompatibility antigen, B alpha chain	P01889	−1.02275
IPO7	Importin-7	O95373	−0.77898
HLA-DRA	HLA class II histocompatibility antigen, DR alpha chain	P01903	−0.71939
eIF3-E	Eukaryotic translation initiation factor 3 subunit E	P60228	−0.67789
STAT5A	Signal transducer and activator of transcription 5A	P42229	−0.60567
RPL30	60S ribosomal protein L30	P62888	−0.58276
SURF4	Surfeit locus protein 4	O15260	−0.55646
eIF3-M	Eukaryotic translation initiation factor 3 subunit M	Q7L2H7	−0.55029
ARF3	ADP-ribosylation factor 3	P61204	−0.52062
NME1	Nucleoside diphosphate kinase A	P15531	−0.51406
(**B**)			
**Gene Name**	**Protein Name**	**Accession**	**Fold-Change**
MIF	Macrophage migration inhibitory factor	P14174	0.491055
IFITM1	Interferon-induced transmembrane protein 1	P13164	0.61364
IFI30	Gamma-interferon-inducible lysosomal thiol reductase	P13284	0.628365
PDLIM1	PDZ and LIM domain protein 1	O00151	0.730311
H2AC4	Histone H2A type 1-B/E	P04908	0.790737
HSPA4L	Heat shock 70 kDa protein 4L	O95757	0.804528
BANF1	Barrier-to-autointegration factor	O75531	0.845222
H1-2	Histone H1.2	P16403	0.847602
DENND4C	DENN domain-containing protein 4C	Q5VZ89	0.855917
(**C**)			
**Gene Name**	**Protein Name**	**Accession**	**Fold-Change**
H1-4	Histone H1.4	P10412	−1.94501
GSPT1	Eukaryotic peptide chain release factor subunit 3a	P15170	−0.97709
H1-2	Histone H1.2	P16403	−0.71206
SUB1	Activated RNA polymerase II transcriptional coactivator p15	P53999	−0.64848
KRT18	Keratin, type I cytoskeletal 18	P05783	−0.56628
ANP32B	Acidic leucine-rich nuclear phosphoprotein 32 family member B	Q92688	−0.55258
H2AC11	Histone H2A type 1	P0C0S8	−0.53413
EIF3CL	Eukaryotic translation initiation factor 3 subunit C-like protein	B5ME19	−0.42143
TPD52L2	Tumor protein D54	O43399	−0.37034
RPL27A	60S ribosomal protein L27a	P46776	−0.36698
(**D**)			
**Gene Name**	**Protein Name**	**Accession**	**Fold-Change**
PSMB2	Proteasome subunit beta type-2	P49721	0.478164
RAB7A	Ras-related protein Rab-7a	P51149	0.485954
NAT10	RNA cytidine acetyltransferase	Q9H0A0	0.547291
EIF3E	Eukaryotic translation initiation factor 3 subunit E	P60228	0.582263
NME1	Nucleoside diphosphate kinase A	P15531	0.600471
RAC2	Ras-related C3 botulinum toxin substrate 2	P15153	0.61141
EIF3M	Eukaryotic translation initiation factor 3 subunit M	Q7L2H7	0.616847
COPB1	Coatomer subunit beta	P53618	0.627621
TPD52	Tumor protein D52	P55327	0.706644
IPO7	Importin-7	O95373	0.820229

Expression values were logarithmized (log2) and the fold change between the two experimental conditions (i.e., culture medium vs. ArtinM treatment) was expressed as a ratio of such (log2) expression values, taking the control condition (culture medium alone) as reference.

## Data Availability

All data presented this study are available from the corresponding author upon responsible request.
